# Association between serum albumin and 60-day mortality in Chinese Hakka patients with non-APL acute myeloid leukemia: a retrospective cohort study

**DOI:** 10.1186/s12885-022-10231-0

**Published:** 2022-11-03

**Authors:** Zuomiao Xiao, Haibo Li, Dejun Xiao, Yulan Liu, Xianchun Chen, Shi Luo, Yanhong Ji

**Affiliations:** 1 Department of Immunology & Microbiology, School of Medicine, Xi’an Jiaotong University, Xi’an, Shaanxi 710049 People’s Republic of China; 2 Department of Clinical Laboratory, The Affiliated Ganzhou Hospital of Nanchang University, Ganzhou, Jiangxi 341000 People’s Republic of China; 3 Division of Birth Cohort Study, Fujian Maternity and Child Health Hospital, Affiliated Hospital of Fujian Medical University, Fuzhou, 350001 People’s Republic of China

**Keywords:** Acute myeloid leukemia, Early mortality, 60-day mortality, Serum albumin

## Abstract

**Background:**

Acute myeloid leukemia (AML) is the main type of adult leukemia, and 60-day mortality is a vital clinical problem that doctors have to face at the begin with treatment. Studies on the association between serum albumin and 60-day mortality from AML (non-APL) are limited.

**Methods:**

In this retrospective cohort study, ALB was measured after admission in all patients diagnosed with primary AML from Affiliated Ganzhou Hospital of Nanchang University between January 2013 and May 2021. The outcome was all-cause, 60-day mortality. Multivariable Cox regression analyses were performed to calculate the adjusted hazard ratio (HR) and its corresponding 95% confidence interval (CI).

**Results:**

This study included 394 primary AML patients. The overall 60-day mortality was 28.9% (114/394); it was 43.1% (56/130), 27.5% (36/131), and 16.5% (22/133) for ALB quantile1 (Q, < 34.5 g/L), quantile 2 (Q2, 34.5–38.5 g/L), and quantile 3 (Q3, ≥ 38.6 g/L), respectively (*P* = 0.001). After adjusting for potential confounders, we found an association between a 6% decrease in 60-day mortality rate and a 1 g/L increase in ALB level (HR = 0.94, 95% CI: 0.89–0.99, *P* = 0.015), which was associated with 38 and 70% decreases in 60-day mortality rates in Q2 (HR = 0.50, 95% CI: 0.30–0.86, *P* = 0.012) and Q3 (HR = 0.47, 95% CI: 0.2 5–0.90, *P* = 0.022), respectively, compared with that in Q1. Similar results were obtained after subgrouping based on an ALB level of 35 g/L (HR = 0.55, 95% CI: 0.34–0.88, *P* = 0.013).

**Conclusions:**

Serum albumin was significantly associated with 60-day mortality of primary AML, which has important clinical significance. Further investigation is warranted.

**Supplementary Information:**

The online version contains supplementary material available at 10.1186/s12885-022-10231-0.

## Introduction

Acute myeloid leukemia (AML) is a heterogeneous clonal myeloid neoplasm characterized by maturation arrest of hematopoietic progenitor cells, leading to uncontrolled blast proliferation. The abnormal differentiation of myeloid cells results in a high level of immature malignant cells and fewer differentiated red blood cells and platelets [1–3]. AML is classified as acute promyelocytic leukemia (APL) and non-APL based upon treatment regimens [4, 5]. 60-day mortality, commonly known as early mortality, defined as death from any cause within 60 days of hospitalization [6–8], is a vital clinical problem, which hematologists are managing to avoid it [9]；Previous studies paid close attention to early mortality in APL, but much less to non-APL, especially in Hakka population. Ganzhou, situated in the southern part of Jiangxi Province, is home to an important Hakka population in China, with a population of nearly 10 million, or 10% of the world’s Hakka population [10]. Serum albumin, a routine laboratory item，is often used clinically to judge the nutritional status or physical condition of patients [11]. This study aimed to assess the association between serum ALB and 60-day mortality among the Chinese Hakka patients with primary AML (non-APL).

## Materials and methods

### Study design and participants

The present retrospective single-center analysis included data of consecutive patients who were primary diagnosed with AML at Affiliated Ganzhou Hospital of Nanchang University (Jiangxi Province, China) between January 1, 2013, and May 31, 2021. All participants in this study underwent bone marrow (BM) aspiration, and AML diagnosis was confirmed based on two or more ways of morphology, immunology, cytogenetic and molecular (MICM) analysis, according to the World Health Organization (WHO) classification system (version 2016) [12]. This study followed the principles of the Declaration of Helsinki and was approved by the Ethics Review Board of Affiliated Ganzhou Hospital of Nanchang University. Given the retrospective nature of the study and the use of anonymous patient data, the requirement for obtaining informed consent was waived [10].

Patients diagnosed APL were excluded because their management and treatment were quite different from the patients with the other subtype of AML [4, 5]. Patients with a history of other hematological malignancies, such as myelodysplastic syndrome (MDS) or myeloproliferative neoplasms (MPN) e.g., were excluded due to secondary AML，because the level of serum ALB may be affected by the primary disease. Individuals with mixed phenotype acute leukemia (MPAL) and AML-M_6_, who did not meet the WHO classification criteria (version 2016), were also excluded as they were categorized as non-AML patients from a strict sense [12]. Patients with liver failure or nephrotic syndrome that causes hypoalbuminemia were excluded from the study.

**Fig. 1:**
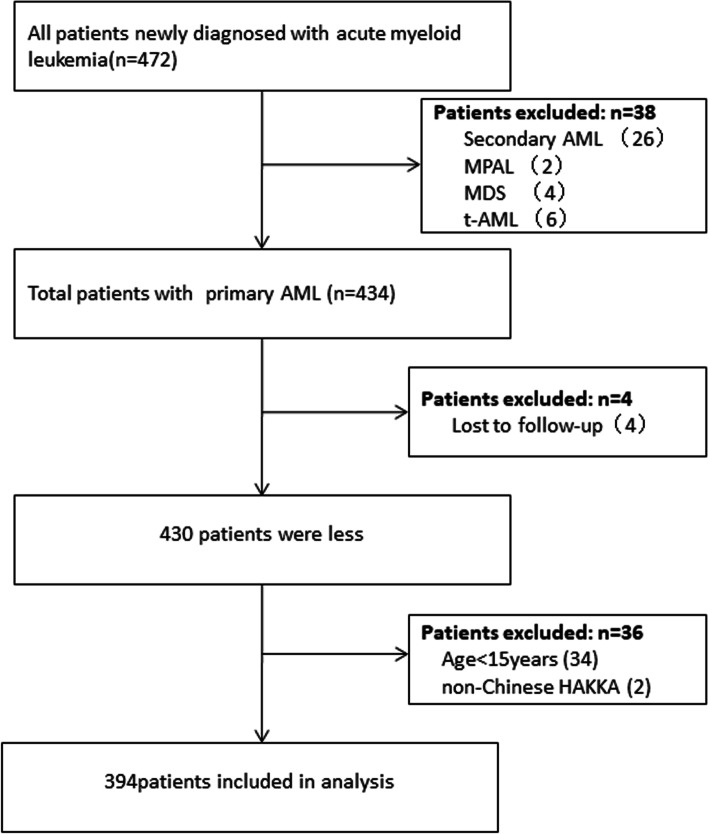
Flowchart of the patient selection process. Abbreviations: *AML*, acute myeloid leukemia; *MPAL*, mixed phenotype acute leukemia; *MDS*, myelodysplastic syndrome; *t-AML*, therapy-related AML

In addition, patients who were aged < 15 years or non-Hakka, did not undergo a serum ALB test in 48 hours of admission, or were lost to follow-up were also excluded; The remaining patients with or without chemotherapy were included in the study. AML patients were divided into four subtypes: AML-M_2_, AML-M_4_, AML-M_5,_ and other subgroups in this cohort study. The flowchart of the patient selection process is presented in Fig. 1.

### Source of data

Data, including survival status, were collected from the electronic medical record system or via follow-up telephone calls. The baseline examinations included blood and BM parameters. The biomarkers included ALB, glucose (Glu), direct bilirubin (DBIL), creatine kinase isoenzyme MB, myoglobin (Myo), serum ferritin (SF), fibrinogen (Fib), BM blast e.g. [10]. All laboratory data included measurements performed within the first 48 hours of admission to reduce the probability that serum biomarker levels were affected by anti-leukemia therapy. These parameters comprise routine testing, commonly used to evaluate the patient’s physical condition. Chemotherapy was administered within 60 days of hospitalization, and none of the patients received bone marrow transplantation.

### Serum ALB

Serum ALB levels were measured using a biochemical analyzer (AU5800. Beckman Coulter, Inc.) with an albumin determination reagent kit (Bromocresol Green method) (Zhejiang Elekon Biotechnology Co., Ltd), read our previous report [10], in brief. The reference interval for ALB was 35–55 g/L, followed the national standard. Every day, we did internal quality control (IQC) (Bio-Rad Laboratories, Inc. Hercules, CA, USA) with commercially available control materials before testing blood samples. Serum ALB was included in the activities of external quality control (EQA), which was hosted by the National Center for Clinical Laboratories (NCCL) thrice a year, and during this study all criteria for feedback reports were fulfilled. All data from this study, including ALB and other items or parameters, were the preliminary test results of patients after admission due to AML. ALB levels were divided into three groups based on ALB quantiles or two based on its level of 35 g/L.

### Outcome

The primary endpoint and outcome of interest were death within 60 days of admission. Collectors of patients’ clinical information at the first diagnosis were blinded to the survival data.

### Statistical analysis

This study aimed to observe the impact of ALB on 60-day mortality in patients with AML. The patients were divided into three groups based on ALB quantiles. A descriptive analysis was asked to all participants. Continuous data were expressed as mean and standard deviation or median and interquartile range ([IQR], quartile 1–quartile 3), as appropriate. The categorical variables were expressed as proportions (%). The variables were compared using of chi-square test (categorical variables), one-way analysis of variance (normal distribution), and Kruskal-Wallis (skewed distribution) tests.

Multivariate Cox regression analysis was used to assess the independent association between serum ALB levels and 60-day mortality. An extended Cox model approach was used for models that were adjusted for various covariates. Covariables were chosen on the basis of previous findings and clinical constraints. Or we adjusted for variables, of which the *p*-values were less than 0.005 for univariate analysis. Survival curves were plotted using of Kaplan-Meier method and were evaluated for statistical significance using log-rank tests. Subgroup analyses were stratified based on the relevant effect covariates. Dummy variables were used to indicate the missing covariancevalues if the missing data variables were greater than 10%. Analyses were stratified according to the results of the univariate analysis (*p* < 0.005), including sex, age, Glu, Myo level, and chemotherapy, to examine the effect of these factors on the above associations. The likelihood ratio test was used to assess the effect modification according to the respective subgroups using interaction terms between subgroup indicators and ALB. Interactions across subgroups were tested using the likelihood ratio test. All analyses were performed using R 3.3.2 (http://www.R-project.org. The R Foundation) and Free Statistics (version 1.4). Differences with a two-sided *P*-value of < 0.05 were considered significant [13].

## Results

### Baseline characteristics of the study participants by categories of serum ALB levels

The final cohort included 394 patients (Fig. 1). None of them had the history of liver failure or nephrotic syndrome. In this study population, 174 patients (44.2%) with AML-M_2_ had the highest proportion, followed by 127 patients (32.2%) with AML-M_5_. The other subgroups were AML-M_0_ (*n* = 4), AML-M_1_ (*n* = 25), AML-M_6_ (*n* = 2), and AML-M_7_ (*n* = 8). The patient’s baseline characteristics are presented in Table 1. The age of the patient was 55.1 ± 17.3 years (range, 15–94 years), 206 (52.3%) were men. A total of 114 patients died within 60 days after admission, including 22 cases of organ failure (respiratory failure 9 cases, heart failure 9 cases, acute renal failure 4 cases), 18 cases of hemorrhagic disease (cerebral hemorrhage 9 cases and gastrointestinal tract 2 cases, DIC 5 cases and others 2 cases), 67 cases of infectious diseases (lung 55 cases, sepsis 6 cases, others 6 cases), There were 7 cases of other causes.

**Table 1:** Baseline patient characteristics according to serum ALB level in primary AML patients

Variables	Total	Serum ALB			*P*-value
		Q1(< 34.6 g/L)	Q2 (34.6–38.5 g/L)	Q3 (≥38.6 g/L)	
Participants, n	394	130	131	133	
Sex, n (%)					0.054
Male	206 (52.3)	79 (60.8)	61 (46.6)	66 (49.6)	
Female	188 (47.7)	51 (39.2)	70 (53.4)	67 (50.4)	
Age	55.1 ± 17.3	60.8 ± 15.1	56.2 ± 15.7	48.5 ± 18.6	< 0.001
ECOG performance-status score, n (%)					0.165
0–1	165 (41.9)	46 (35.4)	61 (46.6)	58 (43.6)	
2–3	229 (58.1)	84 (64.6)	70 (53.4)	75 (56.4)	
Pulmonary infection, n (%)					0.087
No	165 (41.9)	46 (35.4)	64 (48.9)	55 (41.4)	
Yes	229 (58.1)	84 (64.6)	67 (51.1)	78 (58.6)	
FAB subtype, n (%)					0.104
AML-M2	174 (44.2)	48 (36.9)	67 (51.1)	59 (44.4)	
AML-M4	54 (13.7)	15 (11.5)	16 (12.2)	23 (17.3)	
AML-M5	127 (32.2)	54 (41.5)	34 (26.0)	39 (29.3)	
Others	39 (9.9)	13 (10.0)	14 (10.7)	12 (9.0)	
Genomic risk category, n (%)					0.605
Low	53 (18.2)	18 (21.4)	14 (14.3)	21 (19.1)	
Medium	135 (46.2)	40 (47.6)	44 (44.9)	51 (46.4)	
High	104 (35.6)	26 (31)	40 (40.8)	38 (34.5)	
Chemotherapy, n (%)					0.001
No	97 (24.6)	41 (31.5)	38 (29.0)	18 (13.5)	
Yes	297 (75.4)	89 (68.5)	93 (71.0)	115 (86.5)	
Hb (g/L)	68.6 ± 20.4	65.1 ± 17.6	69.4 ± 19.2	71.3 ± 23.6	0.042
Plt (× 10^9^/L)	38.5 (18.2, 70.0)	40.5 (18.0, 68.5)	42.0 (20.5, 78.5)	35.0 (20.0, 68.0)	0.777
INR	1.2 ± 0.2	1.2 ± 0.2	1.1 ± 0.1	1.1 ± 0.3	< 0.001
Fib (g/L)	3.6 ± 1.4	3.7 ± 1.5	3.5 ± 1.4	3.4 ± 1.3	0.268
TBIL (umol/L)	11.8 (8.4, 16.5)	11.2 (7.5, 17.1)	11.4 (8.3, 16.3)	12.1 (9.1, 17.4)	0.211
DBIL (umol/L)	3.7 (2.6, 5.4)	4.0 (2.6, 6.3)	3.3 (2.7, 5.3)	3.5 (2.6, 5.0)	0.194
AST (U/L)	23.1 (16.6, 35.0)	24.0 (16.0, 37.0)	22.0 (16.9, 37.0)	24.0 (16.9, 30.0)	0.509
ALT (U/L)	17.9 (12.0, 29.5)	18.4 (11.8, 31.0)	17.8 (11.9, 31.0)	16.9 (12.0, 27.0)	0.689
Crea (umol/L)	69.0 (56.2, 89.0)	72.5 (59.0, 95.2)	65.1 (54.5, 87.3)	68.3 (57.2, 84.0)	0.084
Glu (mmol/L)	6.5 ± 2.2	6.8 ± 2.6	6.3 ± 2.0	6.4 ± 2.2	0.255
UA, (mmol/L)	346.9 ± 160.2	353.2 ± 171.3	320.5 ± 147.4	366.4 ± 158.8	0.057
BM Blast (%)	57.4 ± 22.2	58.5 ± 22.2	58.1 ± 22.8	55.7 ± 21.5	0.536
SF (ng/mL)	660.8 (387.4, 1218.0)	854.0 (557.1, 1476.0)	569.1 (379.8, 1075.0)	617.7 (294.6, 995.1)	< 0.001
Myo (ng/mL)	20.0 (17.9, 34.1)	25.4 (20.0, 47.2)	20.0 (17.2, 29.8)	20.0 (16.4, 25.1)	0.001
ALB (g/L)	36.4 ± 5.0	30.8 ± 2.8	36.5 ± 1.1	41.8 ± 2.5	< 0.001
60-day mortality, n (%)					< 0.001
No	280 (71.1)	74 (56.9)	95 (72.5)	111 (83.5)	
Yes	114 (28.9)	56 (43.1)	36 (27.5)	22 (16.5)	

Note: data presented are mean ± SD, median (Q1-Q3), or N (%)

*Abbreviations: ALB* albumin; *ECOG* Eastern Cooperative Oncology Group; *FAB* French, American, British; *Hb* hemoglobin; *Plt *platelet; *INR* international normalized ratio; *Fib* Fibrinogen; *TBIL* total bilirubin; *DBIL* direct bilirubin; *AST* aspartate aminotransferase; *ALT* alanine aminotransferase; *Crea* creatinine; *Glu* glucose; *UA* uric acid; *BM* bone marrow; *SF* Serum ferritin; *Myo* myoglobin

In addition, 297 (75.4%) patients received chemotherapy (a combination of cytarabine and anthracycline “7 + 3” or a combination of cytarabine and the other) [14]. A total of 97 patients (24.6%) did not receive chemotherapy, including 67 cases (69.07%) over 60 years old, 10 cases (10.31%) died within 2 weeks after admission, and the others 20 cases (20.62%). The median serum ALB level was 36.4 g/L (range, 21.9–49.2 g/L), and quantile1 (Q1, range, 21.9–34.4 g/L), quantile2 (Q2, range, 34.5–38.5 g/L), quantile1 (Q3, range, 38.6–49.2 g/L). ALB levels decreased with age, and the 60-day mortality increased as the ALB levels decreased (*p* < 0.001).

### Association between serum ALB and 60-day mortality

**Fig. 2:**
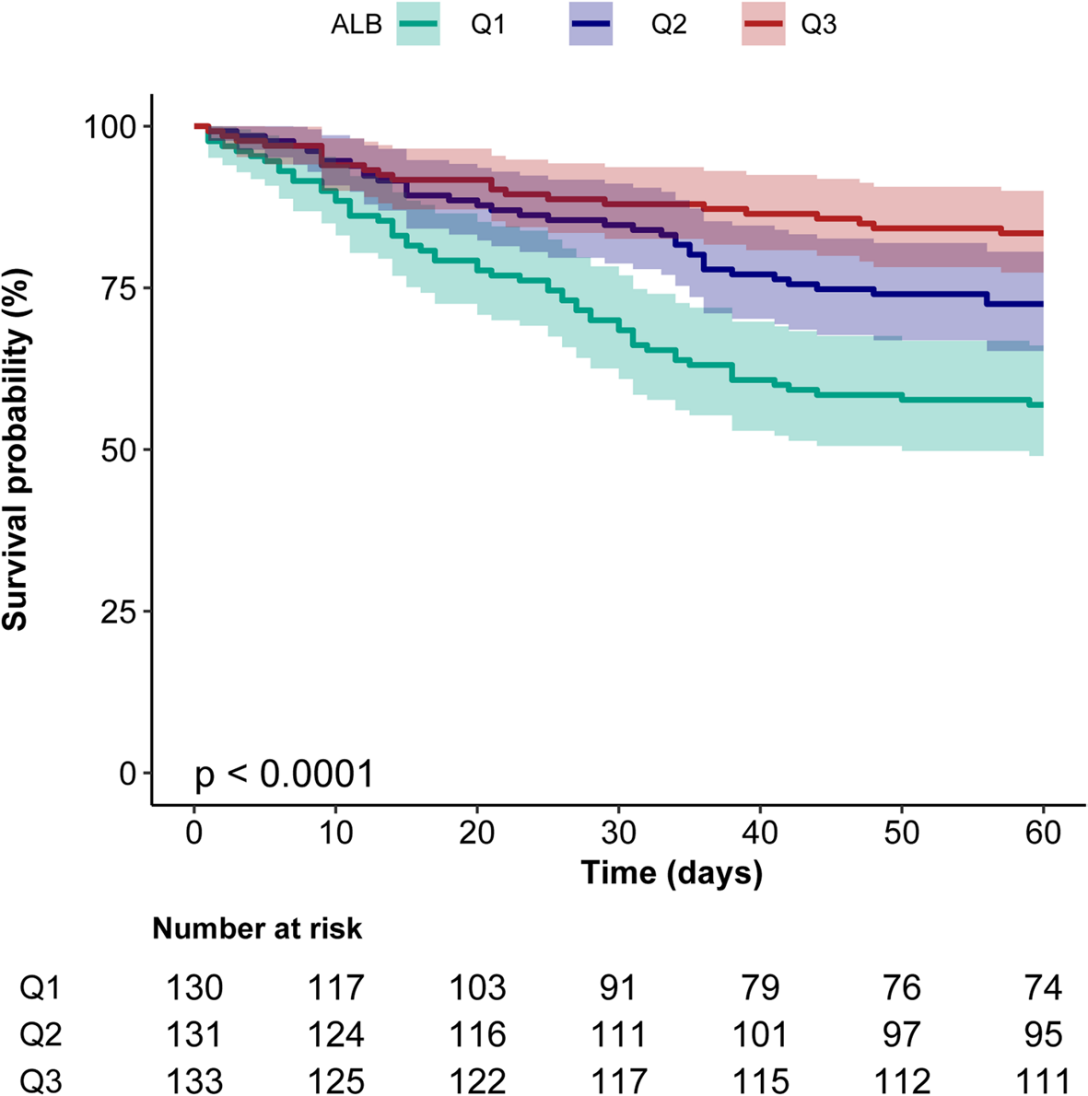
Kaplan-Meier survival curves for day 60 of patients with AML

The Kaplan-Meier curve showed that patients in the ALB Q1 group showed the highest 60-day mortality rate, while those in the ALB Q3 group showed the lowest 60-day mortality rate (log-rank test: *p* < 0.0001, Fig. 2). In the extended multivariable Cox models (Table 2), the hazard ratios (HRs) of serum ALB (per 1 g/L increase) were consistently significant in all three models (HR range: 0.91–0.94). The covariates were selected with univariate analysis of risk factor (attachment Table S1). There were two dummy variables as covariance values, including Genomic risk category (101(25.63%) data missing) and SF (57(14.47%) data missing). After adjustment for all covariance, patients with ALB Q3 demonstrated a 53% decrease in 60-day mortality rate (HR = 0.47, 95% CI: 0.25–0.90, *p* = 0.022, model III), compared with ALB Q1. Similar results were observed when the ALB was divided into two groups based on an ALB level of 35 g/L (HR = 0.55, 95% CI: 0.34–0.88, *p* = 0.013, model III).

**Table 2:** Multivariate Cox regression for ALB on 60-day mortality of AML

Variable	Non-adjusted Model		Model I		Model II		Model III	
	HR (95% CI)	*P-*Value	HR (95% CI)	*P-*Value	HR (95% CI)	*P-*Value	HR (95% CI)	*P*-Value
Serum ALB	0.91 (0.88 ~ 0.94)	< 0.001	0.93 (0.89 ~ 0.96)	< 0.001	0.92 (0.88 ~ 0.97)	0.001	0.94 (0.89 ~ 0.99)	0.015
Binary variable								
ALB< 35 g/L	Ref.		Ref.		Ref.		Ref.	
ALB≥35 g/L	0.47 (0.33 ~ 0.69)	< 0.001	0.59 (0.41 ~ 0.85)	0.005	0.57 (0.37 ~ 0.90)	0.015	0.55 (0.34 ~ 0.88)	0.013
Serum ALB quintile								
Q1 (< 34.6 g/L)	Ref.		Ref.		Ref.		Ref.	
Q2 (34.6–38.5 g/L)	0.56 (0.37 ~ 0.85)	0.006	0.64 (0.42 ~ 0.98)	0.038	0.57 (0.34 ~ 0.94)	0.038	0.50 (0.30 ~ 0.86)	0.012
Q3 ≥ 38.5 g/L)	0.32 (0.20 ~ 0.53)	< 0.001	0.44 (0.27 ~ 0.74)	0.002	0.40 (0.22 ~ 0.73)	0.002	0.47 (0.25 ~ 0.90)	0.022
P for trend		< 0.001		0.001		0.002		0.010

Notes: Model I: Adjusted for sex + age; Model II: adjusted for Model I + Glu + TBIL+ Myo + INR+ Fib+ SF + BM Blast+ FAB subtype+ Chemotherapy; Model III: Adjusted for Model II+ AST+ ALT+ Crea+ UA+ Hb + PLT + Genomic risk category + pulmonary infection+ EGOC performance-status score

*Abbreviations:* *HR* hazard ratio; *CI* confidence index; *ALB* albumin; *Glu* glucose; *TBIL* total bilirubin; *Myo* myoglobin; *INR* international normalized ratio; *Fib* Fibrinogen; *SF* Serum ferritin; *BM *bone marrow; *FAB* French, American, British; *AST* aspartate aminotransferase; *ALT* alanine aminotransferase; *Crea* creatinine; *UA* uric acid; *Hb* hemoglobin; *Plt* platelet; *ECOG* Eastern Cooperative Oncology Group

### Subgroup analyses

**Fig. 3:**
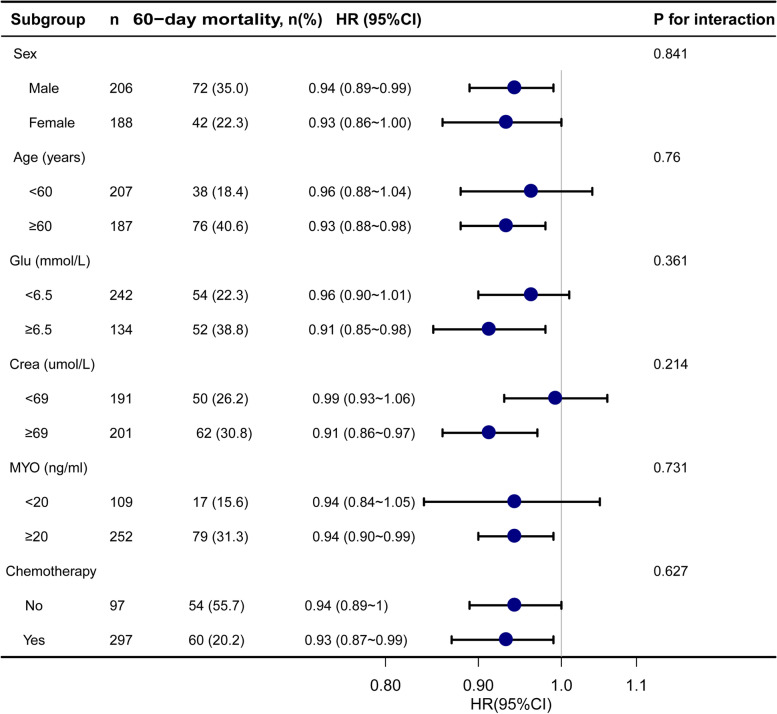
Stratified multivariable analysis of the association between serum ALB and 60-day mortality according to baseline characteristics. Notes: Each stratification adjusts for all factors (age, sex, Glu level, Crea, MYO, and chemotherapy) except for the stratification factor itself. Abbreviations: *AML*, acute myeloid leukemia; *ALB*, albumin; *Glu*, glucose; Crea, creatinine; *MYO*, myoglobin

To detect whether the association between serum ALB levels and 60-day mortality of AML was present in different subgroups, analyses and interactive analyses were stratified according to the confounders, including age, sex, Glu level, MYO level, and chemotherapy (Fig. 3). No significant interactions were observed in the subgroups (All *p*-value for interaction more than 0.05).

## Discussion

Sixty-day mortality of AML still is vital clinical problem cared by hematologist, our study explored the association between serum ALB and 60-day mortality only in patients with primary AML. This study indicates patients with normal serum ALB levels have a lower risk of 60-day mortality, and the risk decreased with an increase in serum ALB levels, regardless of sex, age, Crea level, ECOG performance-status score, chemotherapy, e.g. The results remained robust with no or gradual adjustments.

Previous studies have examined the relationship between serum albumin and survival in AML. Wang et al. examined the association between baseline serum ALB and overall survival (OS) in 243 AML patients (including those with primary and secondary AMLs) who received induction chemotherapy treatment; their results showed that ALB (per 1 g/L increase) were associated with a 9% increase in the OS rate (HR = 0.910, 95% CI: 0.878–0.943), and patients with an ALB level of > 35 g/L had an increasing OS rate of 65.7% compared with those ≤35 g/L (HR = 0.343, 95% CI: 0.241–0.48) [15]; The results of other studies were similar to the findings of our study [16, 17]. However, our study had a larger sample of patients with primary AML, since the secondary ones might have treatment-related complications and low baseline serum albumin levels, due to primary disease or chemotherapy. Furthermore, serum albumin was divided into two or three groups, to explore their association between 60-day mortality, respectively; All the results showed that serum ALB was a protective factor against 60-day mortality in patients with AML, and the protective effect became more significant as the ALB level increased. Serum albumin is a well-known surrogate of the general condition and nutritional status of comorbidities (including liver and kidney function). Serum ALB maintains the normal nutritional state of the human body and colloidal osmotic pressure, such as plasma. Furthermore, it is an indicator of chronic inflammation [6, 7, 18–20]. Low serum albumin levels resulting from inflammation-induced capillary leakage or disease related anorexia during acute illness are associated with poor outcomes [11, 21]. Additionally, Dylan et al. reports that albumin is a major antiapoptotic signaling component and is involved in the transport and metabolism of chemotherapeutic drugs for leukemia [22]. These previous studies may help proving the association between low serum albumin and high 60-day mortality.

Hematologists usually evaluate the risk of early mortality based on the clinical performance status and laboratory data. However, the definition of early mortality in AML remains controversial; it was defined as death within 60 days from the final diagnosis or the start of chemotherapy [6, 7]. Either 60-day mortality or early mortality remains a major clinical problem, which is the first stage toward successful treatment of AML patients. Its causes remain complicated and unclear, even though hematologists have been striving to reduce its risk. The early mortality was 21.0–37.5% reported in previous studies [6, 7, 23, 24], and 60-day mortality was 28.9% in our study, therefore, our result was similar to previous studies. However, our study differs slightly from theirs. Most previous studies only included patients who received chemotherapy [15, 25], while others without chemotherapy were excluded. Among these excluded patients, most of them, were extremely poor making them unsuitable to receive anti-leukemia treatment, due to the existing comorbidities [7]. For example, some patients developed secondary diffuse intravascular coagulation (DIC) with severe intracerebral or pulmonary hemorrhage upon admission, and were not in a position to receive chemotherapy as they died quickly; and they were excluded subjectively. Thus, early mortality in these studies might be reduced due to patient selection bias, and our study might reflect a real association between serum ALB and 60-day mortality in the real world.

This study has several noteworthy limitations. First, although it included several key covariance, unmeasured factors may have contributed to the increased risk of adverse events in patients with a low serum albumin. Second, regardless of the fact these findings raised questions regarding the potential risk for 60-day mortality, interpretation of the results is limited by the observational nature of the study; therefore, the study might not provide direct evidence for predicting 60-day mortality in AML patients. Third, this was a retrospective study; data were collected from 2013 to 2021 (over an 8-year period), and some data on the date of death were obtained by telephone follow-up and may be biased. To reduce bias, interviews with at least two or three family members were conducted to determine the exact survival time of the patients. Meanwhile, different batches of ALB reagents affected the test results to some degree. To make the value dependable, internal quality control (IQC), which becomes the integral part of daily work, is required to ensure the result is controlled before testing the clinical specimens. We have participated an external quality assessment (EQA) organized by the NCCL thrice a year to ensure the accuracy of the testing results since the 1990s. Fortunately, all EQA results were satisfied duration of this study. Meanwhile, the instrument must be calibrated twice a year as part of regular maintenance. Therefore, all the testing results were dependable.

## Conclusion

Serum ALB may be associated with 60-day mortality in patients with AML. These results are of great significance. AML patient with serum albumin < 35 g/L, should be closely managed by the hematologist. Further research is needed to confirm and validate these associations.

## Supplementary Information

**Additional file 1: Table S1.** Univariate analysis of risk factor associated with 60-day mortality in patients with AML (DOCX 18 kb).

## Data Availability

The raw data required to reproduce these findings cannot be shared at this time, as the data also form part of an ongoing study. If necessary, some or all the data generated or used during the study are available from the corresponding author upon request.
