# Supplementary material for: Association between serum albumin and 60-day mortality in Chinese Hakka patients with non-APL acute myeloid leukemia: a retrospective cohort study
Source: BMC Cancer. 2022 Nov 3;22:1127. doi: 10.1186/s12885-022-10231-0 (PMC9632129; doi:10.1186/s12885-022-10231-0)
Supplement: Supplementary file 1 — Additional file 1: Table S1. Univariate analysis of risk factor associated with 60-day mortality in patients with AML (DOCX 18 kb). [file 12885_2022_10231_MOESM1_ESM.docx]

| **Table S1.** Univariate analysis of risk factor associated with 60-day mortality in patients with AML | | |
| --- | --- | --- |
| **Variables** | **HR (95%CI)** | ***P*-Value** |
| Sex |  |  |
| Male | Ref. |  |
| Female | 0.58 (0.40,0.85) | 0.005 |
| Age | 1.04 (1.02,1.05) | <0.001 |
| Pulmonary infection | 1.46 (0.99,2.15) | 0.055 |
| ECOG performance-status score, n (%) | |  |
| 0-1 | Ref. |  |
| 2-3 | 1.07 (0.73,1.55) | 0.736 |
| FAB subtype |  |  |
| AML-M_2_ | Ref. |  |
| AML-M_4_ | 0.64 (0.34,1.23) | 0.185 |
| AML-M_5_ | 0.84 (0.54,1.30) | 0.365 |
| Others | 1.62 (0.95,2.77) | 0.077 |
| Genomic risk category, n (%) | |  |
| Low | Ref. |  |
| Medium | 2.4 (1.01,5.71) | 0.048 |
| High | 1.64 (0.66,4.12) | 0.288 |
| Chemothrerapy | 0.27 (0.18,0.39) | < 0.001 |
| Hb | 0.99 (0.99,1.00) | 0.241 |
| Plt | 1.00(0.99,1.00) | 0.132 |
| INR | 1.51 (0.9,2.53) | 0.117 |
| Fib | 0.94 (0.82,1.08) | 0.412 |
| TBIL | 1.02 (1.00,1.03) | 0.006 |
| DBIL | 1.02 (1.01,1.04) | 0.006 |
| AST | 1.00 (1.00,1.00) | 0.032 |
| ALT | 1.0006 (0.9983,1.0029) | 0.602 |
| Crea | 1.00 (1.00,1.00) | < 0.001 |
| Glu | 1.13(1.07,1.19) | <0.001 |
| UA | 1.00 (1.00,1.00) | 0.744 |
| BM Blast | 1.00 (0.99,1.00) | 0.357 |
| SF | 1.00 (1.00,1.00) | 0.008 |
| Myo | 1.00 (1.00,1.06) | < 0.001 |
| ALB | 0.91 (0.88,0.94) | <0.001 |
| **Abbreviations:** HR, hazard ratio; CI, confidence index; ECOG, Eastern Cooperative Oncology Group; FAB, French, American, British; Hb, hemoglobin; Plt，platelet; INR, international normalized ratio; Fib, Fibrinogen; TBIL, total bilirubin; DBIL, direct bilirubin; AST, aspartate aminotransferase; ALT, alanine aminotransferase; Crea, creatinine; Glu, glucose; UA, uric acid; BM, bone marrow; SF, Serum ferritin; Myo, myoglobin; ALB, albumin | | |
